# Methodological and aerobic capacity adaptations of high‐intensity interval training at different altitudes in distance runners: A comprehensive meta‐analysis

**DOI:** 10.14814/phy2.70349

**Published:** 2025-05-01

**Authors:** Sisay Fentaw, Tefera Tadesse, Zerihun Birhanu

**Affiliations:** ^1^ Sport Academy Bahir Dar University Bahir Dar Ethiopia; ^2^ Department of Sport Science Debark University Debark Ethiopia; ^3^ Educational Development and Quality Center University of Global Health Equity Kigali Rwanda

**Keywords:** altitude, endurance running athletes, high‐intensity interval training, hypoxia, training methodology

## Abstract

High‐intensity interval training (HIIT) in hypoxia has demonstrated superior increases in aerobic capacity (VO_2_ max) adaptations, but this has not been explored in distance runners. This study examined the methodological and VO_2_ max adaptations of HIIT under different altitude conditions in distance runners. We searched the PubMed, ProQuest, Europe PMC, ScienceDirect, and Cochrane databases until August 2024. Among the 1183 reviewed studies, six studies were included. The PEDro score determined the methodological quality, and a meta‐analysis was performed using Jamovi software. The results revealed that VO_2_ max improved more in hypoxic (4.4%–13%) HIIT than in normoxic (1%–8.3%) HIIT. The meta‐analysis results revealed that the effect of HIIT under hypoxia on the VO_2_ max overall standardized mean difference (SMD) was 0.68, with a 95% confidence interval [0.3, 1.06], *p* < 0.001. Conversely, the pooled SMD was not related to the type of hypoxia (*p* = 0.4), training status (*p* = 0.36), intervention week (*p* = 0.82), and sex (*p* = 0.32). In conclusion, HIIT under hypoxia achieves a greater VO_2_ max improvement than normoxia. Endurance athletes and coaches could plan to benefit from such training. However, studies on females using different HIIT protocols and participants at different natural altitudes are limited. This review is registered under the PROSPERO CRD42024578473.

## 1 INTRODUCTION

Training intensity plays a critical role in regulating running sessions through the use of the perceived rate of exertion (RPE), maximum heart rate (HRmax), and aerobic capacity (VO_2_ max) or in combination (Seiler & Kjerland, 2006), for optimal positive influence on VO_2_ max (Londeree, 1997). Accordingly, special attention is needed during high‐intensity interval training (HIIT) at altitude to stimulate the required intensity to influence VO_2_ max. HIIT is speed endurance training, which has been considered an essential part of endurance training programs for decades (Seiler & Kjerland, 2006). It is defined as brief intense training interspersed with high‐intensity intervals of work (85%–95% HRmax or ≥90%VO_2_ max) and passive or active recovery (60%–70% HRmax) durations (Buchheit & Laursen, 2013). It efficiently improves performance and highly benefits endurance athletes (Wahl et al., 2010).

Several high‐intensity protocols have been developed by altering the intensity, volume, and recovery duration (Laursen & Jenkins, 2002). This includes the following: (a) sprint interval training (SIT) is a more intense type performed at the maximal or supramaximal intensity of VO_2_ max for 30 s of “all‐out” with 2–4‐min passive recovery, (b) repeated sprint training (RST) is the most intense type performed several sprint repetitions for ≤10‐s work duration at supramaximal VO_2_ max with <60‐s recovery and (c) HIIT called ‘longer HIIT’ training intervals lasts for 2–6 min (consisting of short 1–2 min, moderate 2–3 min and long intervals ≥4 min) and is performed at about or submaximal VO_2_ max (Atakan et al., 2021; Buchheit & Laursen, 2013).

In proportion, HIIT performed at more than 2‐ to 4‐min intervals is popular for improving endurance performance (Buchheit & Laursen, 2013; Helgerud et al., 2007). In particular, longer HIIT improves VO_2_ max, which plays a key role in running performance compared with SIT (Hov et al., 2023). This is due to the ability to train longer durations around submaximal running efficiency or at maximum VO_2_, which triggers oxygen transport and utilization adaptations to VO_2_ max improvement (Buchheit & Laursen, 2013; Laursen & Jenkins, 2002). The nature of HIIT is more intensive beyond the anaerobic threshold, which must recruit fast‐twitch muscle fibers for strength and power development (Erdogmus et al., 2023). In addition, it also results in improved aerobic performance (Sá Filho et al., 2024). Nevertheless, endurance is characterized as sustaining and withstanding fatigue while performing submaximal intensity movement for longer periods due to the elevated VO_2_ max (da Aparecido Silva et al., 2022).

Although a high VO_2_ max is a critical contributor to endurance performance, it is one of several physiological determinants, such as running economy, lactate threshold, and muscle characteristics that collectively influence middle‐ and long‐distance running performance (van der Zwaard et al., 2021). As such, the maximum oxygen delivery and extraction of the body occur during exhaustive exercise (Abut et al., 2016). The evidence suggests that HIIT is more efficient at improving VO_2_ max in a short time than other modalities are (Helgerud et al., 2007; Hov et al., 2023). However, the efficacy of altitude training is not conclusive (Levine, 2013), and studies have demonstrated changes (Tatte et al., 2022) and no differences (Moges et al., 2024) in VO_2_ max.

Altitude training or hypoxic training is an ergogenic aid involving intentional training under natural or simulated hypoxia (Sinex & Chapman, 2015). As the altitude increases, both the VO_2_ max and the partial pressure of oxygen decrease significantly. Compared with normoxia, this physiological stress is produced at natural altitudes, and simulated hypoxic conditions limit the ability to perform high‐intensity training under hypoxia (Levine & Stray‐Gundersen, 1992). Evidence indicates that altitude reduces running speed by approximately 5%–15% (Peltonen et al., 2001).

Several hypoxic protocols have been developed to obtain the opportunity for altitude training or exposure by alternating among various mechanisms of living and training at different altitudes. The three main types are living and training at high altitude (LHTH), high‐altitude living with low‐altitude training (LHTL), and low‐altitude living with high‐altitude training (LLTH) (Girard et al., 2020; Lundby et al., 2012). However, integrating HIIT with altitude is popular and involves either hypobaric hypoxia (decreased atmospheric pressure) or normobaric hypoxia (decreased fraction of inspired oxygen), which are manipulated under the umbrella of artificial altitudes of the LLTH protocol (Girard et al., 2020; Sinex & Chapman, 2015). This is because the LLTH protocol is cost‐effective and requires less time and effort than other protocols do (Millet et al., 2013).

Compared with that of HIIT under normoxia, the efficiency of combined HIIT and hypoxic training is superior despite the individual capacity to induce performance increases (Faiss, Girard, & Millet, 2013; Levine & Stray‐Gundersen, 1997). In the last two decades, combining these training methods has been popularly applied during the preparation periods of training programs. Several studies have shown that integrating hypoxia with HIIT provides significant VO_2_ max adaptations (Brocherie et al., 2015; Faiss, Girard, & Millet, 2013; Faiss, Léger, et al., 2013; Park et al., 2022). Studies conducted on HIIT under hypoxia for more than 2 weeks have shown a VO_2_ max improvement in trained athletes (Czuba et al., 2013; Faiss, Girard, & Millet, 2013; Roels et al., 2005), physically active individuals (Żebrowska et al., 2019), and sedentary populations (Geiser et al., 2001). In contrast, other studies reported no VO_2_ max improvement (Adams et al., 1975; Neya et al., 2007). However, the heterogeneity of the studies, such as the type of hypoxia, number of intervention weeks, training status, sample size, and sex of the participants might have led to these inconsistencies. Moreover, several meta‐analyses have confirmed that combining HIIT with hypoxia yields greater performance gains than performing the same training at normoxia (Hamlin et al., 2018; Huang et al., 2023; Westmacott et al., 2022). In general, having greater training intensity and duration during hypoxia increases performance improvement (Jung et al., 2020).

Nevertheless, former runners such as Hannes Kolehmainen, Paavo Nurmi, and Emil Zatopek demonstrated the development of HIIT routines (Billat, 2001). Most established reviews on the pooled effect of HIIT with different hypoxic protocols have focused on the performance of sedentary participants (Kong et al., 2017), team sports (Hamlin et al., 2018), mixed sports (Westmacott et al., 2022), and mixed populations (Wen et al., 2019). However, studies on running athletes have revealed inconsistent results in terms of VO_2_ max improvement (Dufour et al., 2006; Nakamoto et al., 2016) and no change (Adams et al., 1975; Neya et al., 2007; Porcari et al., 2016) following HIIT under hypoxia.

Consequently, the need to establish and directly address this issue through a systematic review and meta‐analysis consisting only of running athletes is warranted. Therefore, this study aims to synthesize evidence and combine the pooled effects of HIIT with different hypoxic protocols on the aerobic capacity of middle‐ and long‐distance running athletes. The study also aims to identify gaps and provide insights into the combined effect of HIIT and altitude training protocol type, frequency, and intensity to optimize training for coaches and athletes planning to implement such training methods. More specifically, this study attempts to answer the following research questions.

1. Does the pooled effect indicate a significant VO_2_ max improvement in the combined HIIT and hypoxic conditions?
2. How do subgroup differences such as hypoxic type, training status, training week, and sex influence the VO_2_ max of middle‐ and long‐distance athletes?

## 2 METHODS

### 2.1 Protocol

This review was conducted under the preferred reporting items of systematic review and meta‐analysis (PRISMA) guidelines (Table S3), which systematically gather, identify, and analyze literature review data (Liberati et al., 2009). Furthermore, this review is registered under the PROSPERO database with the registration number CRD42024578473.

### 2.2 Eligibility criteria

#### 2.2.1 Inclusion criteria

The population, intervention, comparison, outcome, and study design approach was used as the inclusion criterion for selecting published articles. However, specific criteria were considered to determine the study's eligibility for inclusion. Hence, studies that met the following criteria were chosen for the review:

1. The study participants (males and females) included those who were classified as middle‐ and long‐distance runners from 3000 m to marathon running and who were trained as recreational or amateur athletes, with ages ranging from 18 to 65 years.
2. The intervention in the present study involved at least 2 weeks of similar HIIT protocols, that is, the SIT, RST, and HIIT protocols, which were performed under different hypoxic protocols, that is, the hypobaric and normobaric hypoxia, hypoxia and normoxia, or LHTH and LLTH protocols.
3. Compared with the hypoxic group, the normoxia group was subjected to the same HIIT, SIT, or RST under normoxic conditions.
4. The primary outcome of the study examined was VO_2_ max adaptation.
5. The study designs considered were experimental randomized controlled trials.

#### 2.2.2 Exclusion criteria

Studies were excluded if i. Participant athletes were from swimming, cycling, triathlon, team sports, nonathletes, or clinical populations; or ii. No detailed information on HIIT protocols or altitude exposure methods; iii. HIIT with hypoxia intervention studies with voluntary hypoventilation, blood flow restriction, and supplementations such as air, temperature, or nitrate; iv. Studies that are non‐English articles published in reputable journals and restricted access to original research papers, review articles, case reports, conference abstracts, and study interventions conducted on animals.

### 2.3 Search strategy

A comprehensive literature search of English electronic databases was systematically conducted on PubMed, Science Direct, Europe PMC, ProQuest, and the Cochrane Central Register of Controlled Trials without publication year restrictions to identify literature on the effects of HIIT at different altitude protocols on the aerobic performance of middle‐ and long‐distance runners. The search period was conducted from inception until August 2024, with a relevant keyword search using the conjunction “and”. We also established search alerts for the databases listed above up to January 2025. A combination of these phrases was used in the search: “high‐intensity interval training” AND “altitude/ hypoxia training”, “sprint interval training” AND “altitude/hypoxia training”, “repeated sprint training” AND “altitude/hypoxia training”. While the databases were being searched, the reference lists of the included articles were manually searched for additional relevant studies. Search restrictions were applied to exclude reviews, conference abstracts, theses, and books or book chapters. We included only English‐language original research articles published in peer‐reviewed journals.

### 2.4 Study selection and data extraction

In this review, we used the EndNote citation management system to select and remove duplicates. The first and third authors independently evaluated the abstracts, titles, and complete texts of the studies to determine if they met the inclusion criteria. In the case where there was a dispute over selecting a paper, the second author was consulted for detailed analysis and a final decision.

### 2.5 Data collection process and data items

After the retrieval of selected papers, a data collection form was created via the Cochrane Data Extraction and Assessment Form template. While the first author collected the data, the third author examined the retrieved data. In the case of disagreements, the second author was involved in the resolution. This fundamental detailed information, which included the author name, publication year, study design, participant age, sex, sample size, HIIT protocol (type, duration, intensity, and frequency), hypoxic protocol (type, height, and time), and outcome measures, was extracted from each of the included articles.

### 2.6 Risk of bias and quality assessment of the included studies

While studies met the inclusion criteria, the first and third authors independently evaluated the papers for internal validity via the Physiotherapy Evidence‐based Database (PEDro) scale to allow careful recording as described elsewhere (Physiotherapy Evidence Database, 2018). They are rigorously used to evaluate the trial quality and study bias of the included studies on the basis of the stated criteria (Sherrington et al., 2000) and are objectively used to assess the methodological quality of each eligible original research study (de Morton, 2009).

While referring to each item, either a “Yes” one point is given for a criterion satisfied or a “No” zero point given for a criterion was unsatisfied (Table 1). While the eligibility criteria were not considered, the sum of 10 points reflects the greatest study quality. A study is considered excellent, fair, or poor in quality when its total score is 6–10, 4–5, or 3 or less, respectively. The PEDro evaluation accordingly includes eligibility criteria, randomization, concealed allocation, baseline comparisons, participant blinding, therapist blinding, assessor blinding, adequate follow‐up, intention‐to‐treat analysis, comparisons, and point estimates and variability.

**TABLE 1:** PEDro score summary of the methodological quality assessment of the included studies.

Study	1	2	3	4	5	6	7	8	9	10	11	Score
Levine & Stray‐Gundersen (1997)	Yes	No	No	Yes	No	No	No	No	Yes	Yes	Yes	4/10
Dufour et al. (2006)	Yes	Yes	No	Yes	No	No	No	Yes	Yes	Yes	Yes	6/10
Jung et al. (2020)	Yes	Yes	No	Yes	No	No	No	Yes	No	Yes	Yes	5/10
Park et al. (2022)	Yes	Yes	No	Yes	No	No	No	Yes	No	Yes	Yes	5/10
Nakamoto et al. (2016)	Yes	Yes	No	Yes	Yes	No	No	No	Yes	Yes	Yes	6/10
Neya et al. (2007)	Yes	Yes	No	Yes	No	No	No	Yes	No	Yes	Yes	5/10

### 2.7 Summary of measures

The main physiological determinant factor in endurance performance is VO_2_ max, that is, the primary outcome variable assessed after HIIT under both hypoxic and normoxic altitude conditions (shown in Table 4). The VO_2_ max measurement method in the studies of Levine and Stray‐Gundersen (1997) and Neya et al. (2007) utilized Douglas bags, and the remaining studies employed breath‐by‐breath analysis. The studies of VO_2_ max measurement took place under normoxia (sea level), with the exception of Neya et al. (2007), who conducted the measurements under hypoxia. All the studies assessed VO_2_ max outcomes before and after the intervention.

### 2.8 Data synthesis

The outcome means, variability values, hypoxic conditions, performance level, and sample size data were extracted from the eligible selected studies. Despite insufficient pretest and posttest data, we tried reaching out to the research author for more details (Jung et al., 2020; Park et al., 2022). However, they did not provide the data within the time frame for unspecified reasons. As a result, the study team estimated from the study's figure via reliable computer software (Imagej.net (accessed on 10 August 2024)).

The data are reported as changes in the mean and standard deviation of the training group. The overall pooled data are reported as the standardized mean difference with a 95% CI. Moreover, computations were performed for both groups' VO_2_ max mean change (posttest minus pretest VO_2_ max in mL^−1^.kg.min^−1^), and in some cases, the standard error of the mean (SEM) data was transformed into standard deviation (SD) (SEM $\sqrt{n}$), and used to calculate the standardized mean difference (SMD), which is a common effect size measure among studies (Gallardo‐Gómez et al., 2024). In the case of missing data, we used the following formulae to calculate the study's SD, ∆SD, and pooled SD: pooled $\mathrm{SD}=\frac{x_{2}-x_{1}}{\text{Cohe}n^{\prime }s\;d},$ pooled $\mathrm{SD}=\sqrt{\frac{\left(n_{1}-1\right){\mathrm{SD}}^{2}1+\left(n_{2}-1\right){\mathrm{SD}}^{2}2}{n_{1}+n_{2}-2},}$ where *x*_1_ and *x*_2_ are the pretest and posttest means of the group, respectively, and *n*_1_ and *n*_2_ are the pre‐post sample sizes of the group. The change in the pretest and posttest VO_2_max standard deviation (∆SD) is calculated via $\Delta \sigma =\sqrt{\left(\sigma_{1}^{2}+\sigma_{2}^{2}-\left(2.{\text{corr}}_{.}.\sigma_{1}.\sigma_{2}\right)\right)},$ where σ, is the standard deviation; and corr.; is the correlation coefficient, which is the value of the association between the pretest and posttest outcomes measured in terms of the time effect. A correlation value of 0.96 from Lawler et al. (1988) baseline and posttest altitude intervention VO_2_ max test relationship values were applied.

### 2.9 Statistical analysis

The results of individual studies were combined via Jamovi software (Jamovi 2.5.6 accessed on 15 August 2024) to perform meta‐analysis and meta‐regression via a random effects model and mixed effects model, respectively. The SMD was used as an outcome measure to perform the analysis. The *I* ^2^ statistic was used to quantify the quality of heterogeneity and determine variation in the predicted effect across studies using the restricted maximum likelihood estimator. The heterogeneity among studies for which a value less than 50% was used was classified as low heterogeneity. The statistical values were set at 95% CIs. The publication bias across the studies' effect sizes and sample sizes was assessed via visual inspection of funnel plots. Egger's regression test was also used to detect small sample size bias quantitatively.

## 3 RESULTS

### 3.1 Literature search and selection

**FIGURE 1:**
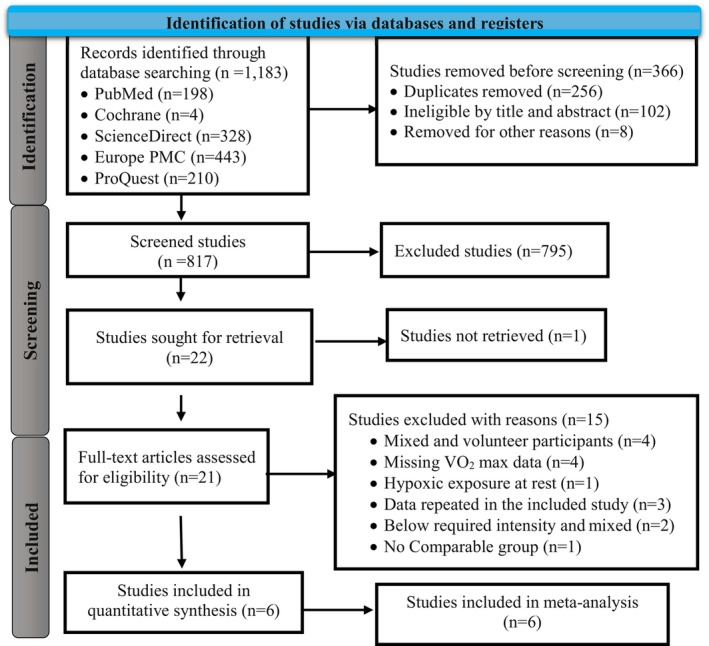
PRISMA flow diagram of the literature search and selection process.

The search strategy was initially conducted on 10 June 2024, and 1183 articles were identified from five electronic databases (see Table S1). However, we excluded fifteen eligible studies after several screening processes for different reasons (see Table S2). The authors reported that only 6 articles met the criteria for analysis (Dufour et al., 2006; Jung et al., 2020; Levine & Stray‐Gundersen, 1997; Nakamoto et al., 2016; Neya et al., 2007; Park et al., 2022). The entire screening process and outcomes are depicted in Figure 1.

### 3.2 Risk of bias and quality within studies

The explicit criteria‐based results of the PEDro score (Table 1) indicate that three studies scored 5/10, one 4/10, and two 6/10 points, with an overall mean score of 5 ± 0.7, where the decision led to fair quality. However, there was no concealed allocation or blinding of the subjects, therapists, or assessors except in Nakamoto et al. (2016) who blinded the participants.

### 3.3 Study characteristics

In the studies by Dufour et al. (2006), Park et al. (2022), and Jung et al. (2020), two comparative groups—hypoxic training groups and normoxic training groups—were used for analysis. While the studies by Levine and Stray‐Gundersen (1997), Neya et al. (2007), and Nakamoto et al. (2016) each included three comparative groups, we selected the groups that aligned with our study objectives. In Levine and Stray‐Gundersen (1997) study, we included the LHTH and LLTL groups while excluding the LHTL group. In Neya et al. (2007), we incorporated the hypoxic training and control groups, excluding the hypoxic exposure‐only group. Similarly, in Nakamoto et al. (2016), we selected intermittent hypoxic training with a normoxic exposure group and normoxic training with a normoxic exposure group (control) while excluding the intermittent hypoxic training with intermittent hypoxic exposure group.

The six studies included 53 males (Dufour et al., 2006; Jung et al., 2020; Neya et al., 2007), 20 females (Park et al., 2022), and 43 participants of both genders (Levine & Stray‐Gundersen, 1997; Nakamoto et al., 2016). All the studies employed randomized controlled trial designs to examine the effects of HIIT under hypoxic and normoxic conditions. In the case of participants' event specialization, studies have recruited subjects from both middle‐ and long‐distance (Jung et al., 2020; Neya et al., 2007) and distance‐running (Dufour et al., 2006; Levine & Stray‐Gundersen, 1997; Nakamoto et al., 2016; Park et al., 2022) events, whose mean ± SD age is 27.6 ± 9 years.

The performance levels of the recruited athletes are as follows: trained (Nakamoto et al., 2016; Neya et al., 2007), moderately trained (Jung et al., 2020), highly trained (Dufour et al., 2006), and well‐trained (Levine & Stray‐Gundersen, 1997; Park et al., 2022). However, we adopted McKay et al. (2022) who proposed training and performance classifications that consolidate the various levels into trained (Jung et al., 2020; Nakamoto et al., 2016; Neya et al., 2007) and highly trained (Dufour et al., 2006; Levine & Stray‐Gundersen, 1997; Park et al., 2022) categories, which show that different training and performance statuses exist among the participants. Consequently, these differences allowed us to determine the effects of HIIT under hypoxia on performance and sex. The complete study characteristics are depicted in Tables 2 and 3.

**TABLE 2:** Descriptive summary of the study and subject methodological characteristics.

Study	Design	Participants			Sport event	Training performance level	Outcome measures
		Group	Number and sex	Age (yrs.)			
Levine & Stray‐Gundersen (1997)	RCT	HTG	13 (9, M; 4, F)	22 ± 3	Distance running	Highly trained	VO_2_ max, hematologic responses, 5000 m TT
		NTG	13 (9, M; 4, F)				
Dufour et al. (2006)	RCT	HTG	9, M	30.3 ± 18.9 30.3 ± 18.3	Distance running	Highly trained	VO_2_ max, T_lim_, VO_2_ kinetics, running economy
		NTG	9, M				
Jung et al. (2020)	RCT	HTG	10, M	26.3 ± 1.5 25.9 ± 1.2	Middle and long‐distance	Trained	VO_2_ max, 3000mTT, BC, hemodynamic, and ANS function
		NTG	10, M				
Park et al. (2022)	RCT	HTG	10, F	24.85 ± 3.8	Distance running	Highly trained	VO_2_ max, 3000mTT, BC, hematology, hemodynamic function
		NTG	10, F				
Nakamoto et al. (2016)	RCT	HTG	10 (2, F; 8, M)	33.3 ± 8.5 42.4 ± 8.5	Distance running	Trained	VO_2_ max, hematology, running economy, lactate threshold
		NTG	7 (3, F; 4, M)				
Neya et al. (2007)	RCT	HTG	9, M	20.0 ± 2.3 20.9 ± 1.5	Middle and long‐distance	Trained	VO_2_ max, total hemoglobin, running economy, 3000mTT
		NTG	6, M				

*Note*: The values are the means ± SDs.

Abbreviations: ANS, autonomic nervous system; BC, body composition; CT, control trial; F, female; HTG, hypoxic training group; M, male; NTG, normoxic training group; RCT, randomized controlled trial; T_lim_, time until exhaustion; TT, time‐trial.

**TABLE 3:** Descriptive summary of maximal aerobic capacity measurement, methodology, and outputs among studies.

Study	*N*	Sport event	VO_2_ max measurement		
			Method	Site	Statistical result
Levine & Stray‐Gundersen (1997)	26	Distance running	Douglas Bag: Modified Astrand‐Saltin protocol	Sea level	↑ sig. at HTG group
Dufour et al. (2006)	18	Distance running	Breath‐by‐breath analysis: ramp protocol	Normoxia	↑ sig. at HTG group
Jung et al. (2020)	20	Middle and long	Breath‐by‐breath analysis: Bruce protocol	Normoxia Chamber	↑sig. in both group
Park et al. (2022)	20	Distance running	Breath‐by‐breath analysis: Bruce protocol.	Normoxia Chamber	↑ sig. in both group
Nakamoto et al. (2016)	17	Distance running	Breath‐by‐breath analysis: ramp protocol	Normoxia	↑ sig. at HTG group
Neya et al. (2007)	15	Middle and long	Douglas Bags: Modified: Astrand‐Saltin protocol	Hypoxia	↔ No sig. change
Total Participants	122				

*Note*: **↑**, statistically improved, **↔**, no significant change.

Abbreviations: N, number of participants; VO_2_ max, aerobic capacity.

With respect to the training protocol, all the studies manipulated HIIT protocols in both the hypoxic and normoxic groups (shown in Table 4). The studies included only a long HIIT protocol with an interval bout duration of 5–30 min and work intensity bouts between 90% and 95% HRmax. The studies used different intermittent hypoxic HIIT intervention durations: two (Nakamoto et al., 2016), four (Levine & Stray‐Gundersen, 1997; Neya et al., 2007), and six (Dufour et al., 2006; Jung et al., 2020; Park et al., 2022) intervention weeks. The altitude conditions used for the hypoxic training group included two types of hypoxia: hypobaric hypoxia using hypobaric chambers (Jung et al., 2020; Park et al., 2022), natural altitude (Levine & Stray‐Gundersen, 1997), and normobaric hypoxia using a nitrogen chamber (Dufour et al., 2006; Nakamoto et al., 2016; Neya et al., 2007). Thus, different methodologies can be used to observe random effects on the basis of intervention duration and altitude.

**TABLE 4:** Descriptive summary of the methodological applications of high‐intensity training and hypoxic conditions.

Study	Group	Altitude/hypoxic condition					HIIT protocol		
		Protocol/group selected/condition	Height in meters	Device	Pb in mm Hg	FiO_2_%	Type	Intervention wk./session.wk.^−1^	Session repetition × interval intensity, recovery
Levine & Stray‐Gundersen (1997)	HTG	LHTH/high‐high/Hypobaric natural	2700	NA	NR	~14.3	HIIT	10/NR	Intensity progressive weekly increase up to 95% HR max
	NTG	LLTL/low‐low/sea level	150	NA	NR	~20.6			
Dufour et al. (2006)	HTG	LLTH/intermittent hypoxic/Normobaric	3000	Chamber	NR	14.5	HIIT	6/2	2 × 12 min at wk. 1 and 4, 2 × 16 min at wk. 2 and 5, 2 × 20 min at wk. 3 × 6 at 90% HR max, rest by 2–5 min
	NTG	LLTL/normoxic/sea level	<300	NR	NR	20.9			
Jung et al. (2020)	HTG	LLTH/hypoxic/Hypobaric	3000	Chamber	526	~14.3	HIIT	6/3	10 × 5 min at 90%–95% HR max, 1 min. recovery
	NTG	LLTL/normoxic/sea level	NR	NR	760	~20.8			
Park et al. (2022)	HTG	LLTH/hypoxic/Hypobaric	3000	Chamber	526	~14.4	HIIT	6/3	10 × 5 min of 90%–95% HR max, 1 min recovery
	NTG	LLTL/normoxic/sea level	NR	NR	760	~20.9			
Nakamoto et al. (2016)	HTG	LLTH/Intermittent hypoxic training + normoxic rest/Normobaric	3000	Chamber	NR	16	HIIT	6/2	2 × 12 min at wk. 1 and 4, 2 × 16 min at wk. 2 and 5, 20 min at wk. 3 and 6 at LT‐HR, 5 min recovery at 60% vVO_2_ max
	NTG	LLTL/normoxic training + normoxic rest	760	NR	NR	20.9			
Neya et al. (2007)	HTG	LLTH/intermittent hypoxic/Normobaric	3000	Room	NR	14.4	HIIT	4/3	30 min at an intensity of 80%–90% HR max
	NTG	LLTL/control/sea‐level	60	NR	NR	–			

Abbreviations: ~, estimated using FiO_2_ = 20.9 × (Pb at altitude/Pb at sea level); FiO_2_, inspired fraction of oxygen; HIIT, high‐intensity interval training; HR max, maximum heart rate; HTG, hypoxia training group; LHTH, live high train high; LLTH, live low train high; LLTL, live low train low; LT‐HR, lactate threshold heart rate; min, minutes; NA, not applicable; NR, not reported; NTG, normoxia training group; Pb, barometric pressure; wk, week.

In terms of the altitude protocols employed, the HIIT intervention was manipulated on the LLTH using simulated altitude chambers and LHTH (Levine & Stray‐Gundersen, 1997) at the natural altitude (2700 m) used in the studies, while the LLTL was maintained at sea level (60–760 m). The hypoxic group HIIT sites were subjected to simulated 3000 m hypoxic conditions with a regulated barometric pressure (Pb) to create hypobaric hypoxia and an inspired fraction of oxygen (FiO_2_) to form normobaric hypoxic environments. Consequently, these findings reveal significantly different physiological hypoxic levels and effects on outcome measures.

Moreover, the studies' VO_2_ max values were relative measurements in milliliters of oxygen consumed per kilogram of body weight per minute (mL.kg^−1^.min^−1^). The findings of these studies revealed that the primary VO_2_ max outcome yields diverse results following the intervention. For example, HIIT under hypoxia may (Dufour et al., 2006; Levine & Stray‐Gundersen, 1997; Nakamoto et al., 2016) or may not (Neya et al., 2007) improve VO_2_ max. On the other hand, improvement was indicated in both the hypoxic and normoxic training groups (Jung et al., 2020; Park et al., 2022), with greater mean change gain in the hypoxic training group.

### 3.4 Results of individual studies

The included studies involved 116 middle‐ and long‐distance athletes. Compared with the normoxic groups, the selected five studies reported significant improvement in VO_2_ max following HIIT under hypoxia, whereas Neya et al. (2007) reported no improvement following HIIT in either group. Specifically, the reported results revealed an increase in the VO_2_ max improvement from 4.4% (Levine & Stray‐Gundersen, 1997) to a remarkable 13.6% (Park et al., 2022) and a reduction of approximately −0.3% (Neya et al., 2007) in the hypoxic training groups (Table 5).

**TABLE 5:** Aerobic capacity quantitative adaptations following high altitude/hypoxic and normoxic high‐intensity interval training.

Study	Group	*N*	VO_2_ max (mL.kg^−1^.min^−1^) m ± SD		Mean change		SD change	Cohen's *d*
			Pretest	Posttest	Raw	%	Raw	
Levine & Stray‐Gundersen (1997)	HTG	13	64.2 ± 1.5	67.0 ± 1.5	2.8	+4.36	2.12	
	NTG	13	64.4 ± 1.8	63.7 ± 1.8	−0.7	−1.09	2.55	
Dufour et al. (2006)	HTG	9	64.2 ± 3.6	67.4 ± 1.3	3.21	+5	3.83	2.56, 95% CI = 1.95, 4.45
	NTG	9	61.5 ± 3.3	62.1 ± 1.1	0.6	+1	3.48	0.55, 95% CI = −0.24, 1.44
Jung et al. (2020)	HTG	10	63.2 ± 2.5	67.2 ± 3.2	4	+6.33	4.06	1.01, *p* < 0.001
	NTG	10	65.0 ± 4.1	66.1 ± 2.2	1.1	+1.69	4.65	0.33, 95% CI = −0.257, 2.457; *p* < 0.01
Park et al. (2022)	HTG	10	64 ± 7.57	72.7 ± 7.57	8.7	+13.6	10.7	1.15, 95% CI: 0.19, 2.00, *p* < 0.05
	NTG	10	63 ± 5.16	68.2 ± 5.16	5.2	+8.3	7.3	0.94, 95% CI: 0.02, 1.79
Nakamoto et al. (2016)	HTG	10	56.6 ± 5.5	59.3 ± 8.2	2.7	+4.8	9.87	0.33 (−0.41, 1.07)
	NTG	7	53.8 ± 5.5	51.8 ± 7.6	−2.04	−3.7	9.38	−0.27 (−1.16, 0.61)
Neya et al. (2007)	HTG	9	58.4 ± 4.4	58.2 ± 4.8	−0.17	−0.3	6.51	
	NTG	6	59.8 ± 7.1	56.8 ± 7.2	−2.99	−5	10.11	

Abbreviations: CI, confidence interval; HTG, hypoxic training group; m, mean; mL.kg^−1^.min^−1^, milliliters of oxygen used per kilogram of body weight per minute; NTG, normoxic training group; SD, standard deviation.

On the other hand, the normoxic training groups presented an increase in VO_2_ max from 1% (Dufour et al., 2006) to 8.2% (Park et al., 2022) and a reduction from 1.09% (Levine & Stray‐Gundersen, 1997) to 5% (Neya et al., 2007) following HIIT under normoxia. This implies that both groups of participants experienced diverse results following HIIT under hypoxic and normoxic conditions, as detailed in Table 5. However, the effect size might be influenced by individual variation, such as sex, training status, and training/exposure protocol. Hence, these five studies underscore that HIIT during hypoxia helps improve the VO_2_ max. However, Park et al. (2022) revealed that women might notice even greater gains from this type of training.

### 3.5 Synthesis of results

In terms of intervention, the studies used similar HIIT programs performed at two different intervention sites, that is, normoxia and hypoxia utilizing LLTL and LLTH altitude protocols, respectively. However, Levine and Stray‐Gundersen (1997) utilized the LHTH and LLTL protocols at natural altitudes. These studies employed different mechanisms to determine exercise intensity. While Dufour et al. (2006) determined the training load via a VO_2_ max test taken at each environmental condition, the other studies determined the training intensity in a normoxic environment. This creates a significant variation in load and adaptations between groups and studies. Despite studies employing different durations, the intervention was maintained at a similar training intensity, with an average of 80%–95% HRmax.

Compared with Jung et al. (2020) and Park et al. (2022), who used ten 5‐min work interval bouts and a 1‐min recovery protocol, most of the selected studies utilized longer intervals of two repetitions of 12, 16, and 20 min (Dufour et al., 2006; Nakamoto et al., 2016) which changed every intervention week to 30 min (Neya et al., 2007) long work interval bouts. These interval duration variations may create diverse VO_2_ max improvement results within and between the groups. Consequently, significant improvement was found in the hypoxic groups (Dufour et al., 2006; Levine & Stray‐Gundersen, 1997; Nakamoto et al., 2016) and both groups (Jung et al., 2020; Park et al., 2022).

### 3.6 Meta‐analysis

**FIGURE 2:**
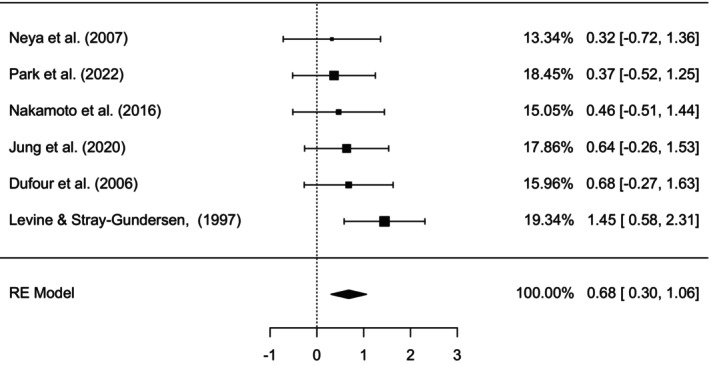
Forest plot for the pooled summary of studies investigating the effect of HIIT under different hypoxic conditions on VO_2_ max arranged by standardized mean difference (SMD) where the individual study name, SMD weighting size (box plots), weight percentage contributing to the pooled SMD, and SMD with 95% CI are set accordingly. The diamond shape indicates the pooled overall SMD using the random effects (RE) model.

A random effects model was used for the meta‐analysis. The test of heterogeneity (*I* ^2^ = 0.00, *p* = 0.5) revealed low heterogeneity. Thus, the SMD for the entire effect of HIIT under hypoxic conditions was 0.68, 95% CI of 0.30–1.06, *p* < 0.001 (Figure 2). In the overall meta‐analysis results of the studies, Levine and Stray‐Gundersen (1997) weighted the most (19.3%), and Neya et al. (2007) held the least (13.3%), which appeared because of the sample size and variance of the studies.

**FIGURE 3:**
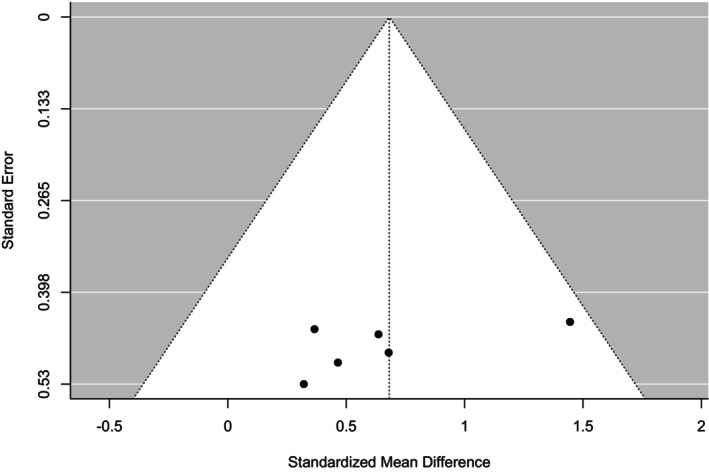
A funnel plot visually determining publication bias on the basis of standardized mean difference and standard error in the included studies investigating the effect of HIIT under different hypoxic conditions on VO_2_ max.

The funnel plot (Figure 3) visually revealed that the included studies were not evenly spread on the plot. Moreover, most values were found on the right‐hand side of the plot. However, the lower part of the plot on the left‐hand side has no indicated study result. This suggests that either null/negative findings are missing or that only positive results are published. Therefore, it is likely asymmetrical, indicating possible publication bias. Neither the rank correlation nor the regression test indicated funnel plot asymmetry (*p* = 0.47 and *p* = 0.23, respectively). This might be due to larger standard errors with smaller sample sizes or heterogeneity between the studies.

However, no studies fell outside where all the studies were inside the funnel. Consequently, we performed a sensitivity analysis by removing the maximum (Levine & Stray‐Gundersen, 1997)‐ and minimum (Neya et al., 2007)‐weighted studies individually and in combination. However, this did not have a significant effect on the direction of the value of the eliminated maximum weight (0.5 [0.08–0.92]), least weight (0.74 [0.33, 1.15]), or combined weight (0.53 [0.07, 1.00]). Moreover, the Begg and Mazumdar rank correlation (*p* = 0.5) and Egger's regression (*p* = 0.2) tests indicated no significant publication bias. The trim and fill methods (2) suggest that two missing studies might have minimal bias but are not sufficient to change conclusions, and fail‐safe N (21, *p* < 0.001) 21 unpunished null results needed to increase the effect size to nonsignificance. This finding indicates a moderately robust influence that may not be a major concern for publication bias, so the meta‐analysis is robust against the analysis.

### 3.7 Meta‐regression

We performed a meta‐regression analysis for study heterogeneity or subgroup differences. Consequently, we ran a mixed‐effects model for the coefficient estimate and 95% CI to examine their influence on the overall SMD. As a result, the effects of the type of hypoxia (0.33, 95% CI [−0.44, 1.09], *p* = 0.4), training status (−0.36, [−1.12, 0.41], *p* = 0.36), intervention week (−0.06, [−0.6, 0.48], *p* = 0.82), and sex (−0.21, [−0.64, 0.21], *p* = 0.32) on the SMD were not related. However, the relationship between the intercept effect size and SMD was insignificant during the meta‐regression analysis except for the sex subgroup (*p* = 0.02). This could be due to the high proportion of representative male sample sizes.

## 4 DISCUSSION

This study aimed to analyze the current evidence on the effects of HIIT under different hypoxic conditions on the VO_2_ max of middle‐ and long‐distance running athletes. Consequently, on the basis of the objectives we set, first, the pooled effect of combined HIIT with hypoxic conditions indicated a significant improvement in VO_2_ max in the hypoxic training group compared with the normoxic training group. Second, subgroup differences, such as type of hypoxia, training status, training week, and sex, were not related to the pooled effect in middle‐ and long‐distance athletes. However, these subgroup analysis results should be interpreted with caution due to the lower number of included studies to conclude. This training is a vogue due to the efficacy of improving aerobic capacity in a short period over other modalities. Accordingly, the pooled evidence of the meta‐analysis indicates that VO_2_ max improvement using HIIT under hypoxia yields greater positive gains than HIIT under normoxia.

However, Dufour et al. (2006) determined the intervention intensity for a hypoxic training group at hypoxia, which induces reductions in training loads and relative intensity due to variations in oxygen access and physiological responses. Consequently, the notable change in performance of athletes may not have been fully realized following the hypoxic determined training intensity. On the other hand, the study by Neya et al. (2007) demonstrated nonsignificant VO_2_ max results in both groups compared with the included studies. These differences may be due to the dose–response of individual variability, time of exposure, or level of hypoxia, while VO_2_ max is measured under hypoxia. Neya et al. (2007) reported that the accumulated total exposure time to hypoxia and the level of hypoxia (360 min and 3000 m) were lower than those reported in the included studies (480–900 min and 2500–3000 m). In addition, the small sample size of the Neya et al. (2007) control group (*n* = 6) may induce a risk of false negatives to detect real significance.

The potential difference between the hypoxic training group and the normoxic training group was the level of hypoxia, in which the former utilized limited Pb or FiO_2_ conditions compared with the latter group, which was trained in favorable sea‐level environments. At altitude, the formation of physiological and environmental factors significantly influences both high‐intensity training and VO_2_ max. This stress led the hypoxic training groups to elicit VO_2_ max adaptations. The VO_2_ max is a primary determining physiological variable that plays a key role in running as the race distance increases (Spencer & Gastin, 2001), especially in middle and long‐distance running performance (Legaz‐Arrese et al., 2007; Morgan & Daniels, 1994). It can be improved more when HIIT is used at the optimal intensity of 95%–100% of the VO_2_ max (Midgley et al., 2006). Considering these benefits, several studies have been conducted to establish a body of knowledge on its efficiency in different populations (Atakan et al., 2021; Brocherie et al., 2015; Huang et al., 2023; Westmacott et al., 2022).

Furthermore, this study demonstrated that HIIT under hypoxic conditions can elicit additional gains in the VO_2_ max of middle‐ and long‐distance running athletes. As a result, high‐intensity training during hypoxia has emerged as a promising training protocol. This study is consistent with previous meta‐analyses that reported a VO_2_ max improvement in the effectiveness of hypoxic protocols in athletes and nonathletes with an SMD ranging from 1.45 to −7.10 (Yu et al., 2023), HIIT at a hypoxia SMD of 1.45 (Westmacott et al., 2022) and intermittent hypoxic training in exercisers, with a weighted mean difference of 3.2 (Huang et al., 2023). These pooled effect sizes indicate a VO_2_ max improvement in response to the hypoxic intervention when the intensity of the VO_2_ max was positive.

However, the study results demonstrated no relationship between the SMD and the stated moderators, which indicates that these factors may not influence aerobic capacity. This may be due to the limited methodological variation among the included studies. For example, the training intensities used in the hypobaric hypoxia (Pb = 526 mmHg) and normobaric hypoxia (FiO_2_ = 14.3 to −16) groups were equivalent. This result is consistent with the findings of Westmacott et al. (2022), who reported the absence of a dose–response relationship. However, Feng et al. (2023) determined the existence of relationships in hypoxic duration and intensity, whereas optimal hypoxic training varies among athletes.

The VO_2_ max is achieved during maximum oxygen utilization of the body in response to prolonged exhaustive exercise (Abut et al., 2016). Although this variable plays a key role in running performance, it has little influence on almost equivalent performance among elite athletes (Snell & Mitchell, 1984). In such cases, efficient running (Conley & Krahenbuhl, 1980) and velocity at VO_2_ max (Schabort et al., 2000) play key roles in running performance. However, a certain amount of VO_2_ max improvement can contribute to winning in a small fraction of a second. The longer interval bout duration of HIIT plus the already formed natural environment challenges under hypoxic conditions would allow the body to respond to physiological and hormonal changes, which directly stimulate and contribute to the development of VO_2_ max.

In the hypoxic groups, the VO_2_ max improved in the included studies compared with the pretest measures. Compared with the baseline measurements, improvements in the normoxic groups were observed, but these improvements were less pronounced than those in the hypoxic groups. Thus, the results of this review revealed that, compared with training under normoxia, HIIT at different altitudes/hypoxic conditions yields additional adaptations in VO_2_ max. This improvement might be due to the ability to train longer durations at approximately submaximal running efficiency or at maximum VO_2_, which triggers adaptations in oxygen transport and utilization, leading to VO_2_ max improvement (Buchheit & Laursen, 2013; Laursen & Jenkins, 2002).

## 5 LIMITATIONS

The inclusion of more studies in terms of study design, training protocols, and outcome measures is limited in this review. Moreover, the studies included were searched in certain freely accessible databases. As a result, some articles may not have the opportunity to be reviewed for robustness and reliable conclusions, which is considered a limitation. Nevertheless, we produced a sufficient number of studies that were considered representative of performing this analysis. The VO_2_ max measurement site for one included study was taken under both conditions. However, we opted for the normoxia measure to establish sites similar to those used in other studies. The PEDro score for study quality was 5, indicating fair quality. Nonetheless, it may be difficult to blind participants to such interventions, but future rigorous research should utilize possible mechanisms of participant blinding.

## 6 CONCLUSION

The results of this comprehensive meta‐analysis suggest that concurrently combining HIIT with a hypoxic environment is an effective strategy for improving VO_2_ max compared with combining HIIT with normoxia in middle‐ and long‐distance running athletes. Despite the absence of associations between the methodological adaptations (type of hypoxia, training status, intervention week, and sex) and aerobic capacity improvement in the hypoxic groups, an insufficient number of representative studies resulted in the detection of nonsignificant associations. Hence, the results of all the selected studies revealed a positive SMD favoring HIIT in the hypoxic training groups compared with the normoxic training groups.

## 7 FUTURE DIRECTIONS

While maintaining the observed results in the reviewed studies, future research efforts should aim to address the limitations of the current evidence. Specifically, studies should be conducted with larger sample sizes, a greater representation of female participants, and various altitudes and HIIT modalities to optimize the performance of middle‐ and long‐distance running athletes. This review confirms that the effect is positive. Nevertheless, some influencing factors, such as training volume and duration of hypoxic training, were not quantified in the analysis. In practical terms, these factors may have potential effects on outcomes, which we recommend for further investigation.

Furthermore, significantly more attention has been given to lower‐altitude athletes in experimental studies. Despite the success of altitude‐native long‐distance athletes, there is a lack of research regarding the effectiveness of combined HIIT under different hypoxic conditions. Compared with their lower‐altitude counterparts, altitude‐native athletes possess distinct physiological characteristics and training adaptations. Consequently, the pooled results represent only the performance of those residing at lower altitudes. Therefore, future research is warranted to clarify whether this training positively contributes to the holistic performance of altitude‐native long‐distance athletes.

The hypoxic training group in the included studies utilized the LLTH protocol in a highly controlled environment of intermittent hypoxic training, which may not apply to real‐life long‐distance training and competition. Therefore, investigating the combined effects of HIIT at natural altitudes on performance measures in either altitude‐native or lower‐altitude middle‐ and long‐distance running athletes is important.

## FUNDING INFORMATION

No funding source was granted.

## CONFLICT OF INTEREST STATEMENT

None.

## ETHICS STATEMENT

This study is a comprehensive meta‐analysis and does not require ethical consent.

## Supporting information


Appendix S1.

## Data Availability

The datasets used and analyzed during the current study are available from the corresponding author upon reasonable request.

## References

[phy270349-bib-0001] Abut, F. , Akay, M. F. , & George, J. (2016). Developing new VO(2)max prediction models from maximal, submaximal and questionnaire variables using support vector machines combined with feature selection. Computers in Biology and Medicine, 79, 182–192. 10.1016/j.compbiomed.2016.10.018 27810624

[phy270349-bib-0002] Adams, W. C. , Bernauer, E. M. , Dill, D. B. , & Bomar, J. B., Jr. (1975). Effects of equivalent sea‐level and altitude training on VO2max and running performance. Journal of Applied Physiology, 39(2), 262–266. 10.1152/jappl.1975.39.2.262 1176388

[phy270349-bib-0003] Atakan, M. M. , Li, Y. , Koşar, Ş. N. , Turnagöl, H. H. , & Yan, X. (2021). Evidence‐based effects of high‐intensity interval training on exercise capacity and health: A review with historical perspective. International Journal of Environmental Research and Public Health, 18(13), 7201. 10.3390/ijerph18137201 34281138 PMC8294064

[phy270349-bib-0004] Billat, L. V. (2001). Interval training for performance: A scientific and empirical practice: Special recommendations for middle‐and long‐distance running. Part I: Aerobic interval training. Sports Medicine, 31, 13–31. 11219499 10.2165/00007256-200131010-00002

[phy270349-bib-0005] Brocherie, F. , Girard, O. , Faiss, R. , & Millet, G. P. (2015). High‐intensity intermittent training in hypoxia: A double‐blinded, placebo‐controlled field study in youth football players. Journal of Strength and Conditioning Research, 29(1), 226–237. 10.1519/jsc.0000000000000590 24978836

[phy270349-bib-0006] Buchheit, M. , & Laursen, P. B. (2013). High‐intensity interval training, solutions to the programming puzzle: Part I: Cardiopulmonary emphasis. Sports Medicine, 43(5), 313–338. 23539308 10.1007/s40279-013-0029-x

[phy270349-bib-0007] Conley, D. L. , & Krahenbuhl, G. S. (1980). Running economy and distance running performance of highly trained athletes. Medicine and Science in Sports and Exercise, 12(5), 357–360. 7453514

[phy270349-bib-0008] Czuba, M. , Zając, A. , Maszczyk, A. , Roczniok, R. , Poprzęcki, S. , Garbaciak, W. , & Zając, T. (2013). The effects of high intensity interval training in normobaric hypoxia on aerobic capacity in basketball players. Journal of Human Kinetics, 39, 103–114. 10.2478/hukin-2013-0073 24511346 PMC3916912

[phy270349-bib-0009] da Aparecido Silva, R. , Leite Rocco, P. G. , Stelmach, R. , da Mara Silva Oliveira, L. , Sato, M. N. , Cukier, A. , & Carvalho, C. R. F. (2022). Constant‐load exercise versus high‐intensity interval training on aerobic fitness in moderate‐to‐severe asthma: A randomized controlled trial. Journal of Allergy and Clinical Immunology: In Practice, 10(10), 2596–2604. 10.1016/j.jaip.2022.05.023 35654369

[phy270349-bib-0010] de Morton, N. A. (2009). The PEDro scale is a valid measure of the methodological quality of clinical trials: A demographic study. The Australian Journal of Physiotherapy, 55(2), 129–133. 10.1016/s0004-9514(09)70043-1 19463084

[phy270349-bib-0011] Dufour, S. P. , Ponsot, E. , Zoll, J. , Doutreleau, S. , Lonsdorfer‐Wolf, E. , Geny, B. , Lampert, E. , Flück, M. , Hoppeler, H. , Billat, V. , Mettauer, B. , Richard, R. , & Lonsdorfer, J. (2006). Exercise training in normobaric hypoxia in endurance runners. I. Improvement in aerobic performance capacity. Journal of Applied Physiology (Bethesda, MD: 1985), 100(4), 1238–1248. 10.1152/japplphysiol.00742.2005 16540709

[phy270349-bib-0012] Erdogmus, T. N. , Aras, D. , Gulu, M. , Aldhahi, M. I. , Dahesh, A. M. , & Badri, S. (2023). Combination of high‐intensity interval training and creatine intake enhances leg strength and anaerobic power without changes in body composition in physically active adult men.

[phy270349-bib-0013] Faiss, R. , Girard, O. , & Millet, G. P. (2013). Advancing hypoxic training in team sports: From intermittent hypoxic training to repeated sprint training in hypoxia. British Journal of Sports Medicine, 47(Suppl 1), i45–i50. 10.1136/bjsports-2013-092741 24282207 PMC3903143

[phy270349-bib-0014] Faiss, R. , Léger, B. , Vesin, J. M. , Fournier, P. E. , Eggel, Y. , Dériaz, O. , & Millet, G. P. (2013). Significant molecular and systemic adaptations after repeated sprint training in hypoxia. PLoS One, 8(2), e56522. 10.1371/journal.pone.0056522 23437154 PMC3577885

[phy270349-bib-0015] Feng, X. , Zhao, L. , Chen, Y. , Wang, Z. , Lu, H. , & Wang, C. (2023). Optimal type and dose of hypoxic training for improving maximal aerobic capacity in athletes: A systematic review and Bayesian model‐based network meta‐analysis. Frontiers in Physiology, 14, 1223037. 10.3389/fphys.2023.1223037 37745240 PMC10513096

[phy270349-bib-0016] Gallardo‐Gómez, D. , Richardson, R. , & Dwan, K. (2024). Standardized mean differences in meta‐analysis: A tutorial. Cochrane Evidence Synthesis and Methods, 2(3), e12047. 10.1002/cesm.12047 PMC11795939 40475185

[phy270349-bib-0017] Geiser, J. , Vogt, M. , Billeter, R. , Zuleger, C. , Belforti, F. , & Hoppeler, H. (2001). Training high‐‐living low: Changes of aerobic performance and muscle structure with training at simulated altitude. International Journal of Sports Medicine, 22(8), 579–585. 10.1055/s-2001-18521 11719893

[phy270349-bib-0018] Girard, O. , Brocherie, F. , Goods, P. S. , & Millet, G. P. (2020). An updated panorama of “living low‐training high” altitude/hypoxic methods. Frontiers in Sports and Active Living, 2, 26. 33345020 10.3389/fspor.2020.00026 PMC7739748

[phy270349-bib-0019] Hamlin, M. J. , Lizamore, C. A. , & Hopkins, W. G. (2018). The effect of natural or simulated altitude training on high‐intensity intermittent running performance in team‐sport athletes: A meta‐analysis. Sports Medicine, 48(2), 431–446. 10.1007/s40279-017-0809-9 29129021

[phy270349-bib-0020] Helgerud, J. , Høydal, K. , Wang, E. , Karlsen, T. , Berg, P. , Bjerkaas, M. , Simonsen, T. , Helgesen, C. , Hjorth, N. , & Bach, R. (2007). Aerobic high‐intensity intervals improve VO2max more than moderate training. Medicine & Science in Sports & Exercise, 39(4), 665–671. 17414804 10.1249/mss.0b013e3180304570

[phy270349-bib-0021] Hov, H. , Wang, E. , Lim, Y. R. , Trane, G. , Hemmingsen, M. , Hoff, J. , & Helgerud, J. (2023). Aerobic high‐intensity intervals are superior to improve V̇O(2max) compared with sprint intervals in well‐trained men. Scandinavian Journal of Medicine & Science in Sports, 33(2), 146–159. 10.1111/sms.14251 36314990 PMC10099854

[phy270349-bib-0022] Huang, Z. , Yang, S. , Li, C. , Xie, X. , & Wang, Y. (2023). The effects of intermittent hypoxic training on the aerobic capacity of exercisers: A systemic review and meta‐analysis. BMC Sports Science, Medicine and Rehabilitation, 15(1), 174. 10.1186/s13102-023-00784-3 PMC10731756 38115083

[phy270349-bib-0023] Jung, W. S. , Kim, S. W. , & Park, H. Y. (2020). Interval hypoxic training enhances athletic performance and does not adversely affect immune function in middle‐ and long‐distance runners. International Journal of Environmental Research and Public Health, 17(6), 1934. 10.3390/ijerph17061934 32188027 PMC7143158

[phy270349-bib-0024] Kong, Z. , Shi, Q. , Nie, J. , Tong, T. K. , Song, L. , Yi, L. , & Hu, Y. (2017). High‐intensity interval training in Normobaric hypoxia improves cardiorespiratory fitness in overweight Chinese young women. Frontiers in Physiology, 8, 175. 10.3389/fphys.2017.00175 28386234 PMC5362639

[phy270349-bib-0025] Laursen, P. B. , & Jenkins, D. G. (2002). The scientific basis for high‐intensity interval training: Optimising training programmes and maximising performance in highly trained endurance athletes. Sports Medicine, 32(1), 53–73. 10.2165/00007256-200232010-00003 11772161

[phy270349-bib-0026] Lawler, J. , Powers, S. K. , & Thompson, D. (1988). Linear relationship between VO2max and VO2max decrement during exposure to acute hypoxia. Journal of Applied Physiology (Bethesda, MD: 1985), 64(4), 1486–1492. 10.1152/jappl.1988.64.4.1486 3378983

[phy270349-bib-0027] Legaz‐Arrese, A. , Munguía‐Izquierdo, D. , Nuviala Nuviala, A. , Serveto‐Galindo, O. , Moliner Urdiales, D. , & Reverter Masía, J. (2007). Average VO2max as a function of running performances on different distances. Science & Sports, 22(1), 43–49. 10.1016/j.scispo.2006.01.008

[phy270349-bib-0028] Levine, B. (2013). 12 Training and exercise at high altitudes. In Sport, Leisure and Ergonomics (p. 74). Taylor & Francis.

[phy270349-bib-0029] Levine, B. D. , & Stray‐Gundersen, J. (1992). A practical approach to altitude training: Where to live and train for optimal performance enhancement. International Journal of Sports Medicine, 13(Suppl 1), S209–S212. 10.1055/s-2007-1024642 1483778

[phy270349-bib-0030] Levine, B. D. , & Stray‐Gundersen, J. (1997). “Living high‐training low”: Effect of moderate‐altitude acclimatization with low‐altitude training on performance. Journal of Applied Physiology (Bethesda, MD: 1985), 83(1), 102–112. 10.1152/jappl.1997.83.1.102 9216951

[phy270349-bib-0031] Liberati, A. , Altman, D. G. , Tetzlaff, J. , Mulrow, C. , Gøtzsche, P. C. , Ioannidis, J. P. , Clarke, M. , Devereaux, P. J. , Kleijnen, J. , & Moher, D. (2009). The PRISMA statement for reporting systematic reviews and meta‐analyses of studies that evaluate health care interventions: Explanation and elaboration. Journal of Clinical Epidemiology, 62(10), e1–e34. 10.1016/j.jclinepi.2009.06.006 19631507

[phy270349-bib-0032] Londeree, B. R. (1997). Effect of training on lactate/ventilatory thresholds: A meta‐analysis. Medicine & Science in Sports & Exercise, 29(6), 837–843. 10.1097/00005768-199706000-00016 9219214

[phy270349-bib-0033] Lundby, C. , Millet, G. P. , Calbet, J. A. , Bärtsch, P. , & Subudhi, A. W. (2012). Does ‘altitude training'increase exercise performance in elite athletes? British Journal of Sports Medicine, 46(11), 792–795. 22797528 10.1136/bjsports-2012-091231

[phy270349-bib-0034] McKay, A. K. A. , Stellingwerff, T. , Smith, E. S. , Martin, D. T. , Mujika, I. , Goosey‐Tolfrey, V. L. , Sheppard, J. , & Burke, L. M. (2022). Defining training and performance caliber: A participant classification framework. International Journal of Sports Physiology and Performance, 17(2), 317–331. 10.1123/ijspp.2021-0451 34965513

[phy270349-bib-0035] Midgley, A. W. , McNaughton, L. R. , & Wilkinson, M. (2006). Is there an optimal training intensity for enhancing the maximal oxygen uptake of distance runners?: Empirical research findings, current opinions, physiological rationale and practical recommendations. Sports Medicine, 36(2), 117–132. 10.2165/00007256-200636020-00003 16464121

[phy270349-bib-0036] Millet, G. P. , Faiss, R. , Brocherie, F. , & Girard, O. (2013). Hypoxic training and team sports: A challenge to traditional methods? (Vol. 47, pp. i6–i7). BMJ publishing group Ltd and British Association of Sport and Exercise Medicine. 10.1136/bjsports-2013-092793 PMC3903151 24282210

[phy270349-bib-0037] Moges, T. , Dhamodharan, M. , Gebretensay, M. , Kiflu, A. , & Kentiba, E. (2024). The effect of altitude training on physiological variables of endurance athletes in Ethiopia. Physical Rehabilitation & Recreational Health Technologies, 9(5), 431–442.

[phy270349-bib-0038] Morgan, D. W. , & Daniels, J. T. (1994). Relationship between VO2max and the aerobic demand of running in elite distance runners. International Journal of Sports Medicine, 15(7), 426–429. 10.1055/s-2007-1021082 8002123

[phy270349-bib-0039] Nakamoto, F. P. , Ivamoto, R. K. , Andrade Mdos, S. , de Lira, C. A. , Silva, B. M. , & da Silva, A. C. (2016). Effect of intermittent hypoxic training followed by intermittent hypoxic exposure on aerobic capacity of long distance runners. Journal of Strength and Conditioning Research, 30(6), 1708–1720. 10.1519/jsc.0000000000001258 26562716

[phy270349-bib-0040] Neya, M. , Enoki, T. , Kumai, Y. , Sugoh, T. , & Kawahara, T. (2007). The effects of nightly normobaric hypoxia and high intensity training under intermittent normobaric hypoxia on running economy and hemoglobin mass. Journal of Applied Physiology (Bethesda, MD: 1985), 103(3), 828–834. 10.1152/japplphysiol.00265.2007 17556496

[phy270349-bib-0041] Park, H. Y. , Jung, W. S. , Kim, S. W. , Kim, J. , & Lim, K. (2022). Effects of interval training under hypoxia on hematological parameters, hemodynamic function, and endurance exercise performance in amateur female runners in Korea. Frontiers in Physiology, 13, 919008. 10.3389/fphys.2022.919008 35665230 PMC9158122

[phy270349-bib-0042] Peltonen, J. E. , Tikkanen, H. O. , & Rusko, H. K. (2001). Cardiorespiratory responses to exercise in acute hypoxia, hyperoxia and normoxia. European Journal of Applied Physiology, 85(1–2), 82–88. 10.1007/s004210100411 11513325

[phy270349-bib-0043] Porcari, J. P. , Probst, L. , Forrester, K. , Doberstein, S. , Foster, C. , Cress, M. L. , & Schmidt, K. (2016). Effect of wearing the elevation training mask on aerobic capacity, lung function, and hematological variables. Journal of Sports Science and Medicine, 15(2), 379–386. 27274679 PMC4879455

[phy270349-bib-0044] Roels, B. , Millet, G. P. , Marcoux, C. , Coste, O. , Bentley, D. J. , & Candau, R. B. (2005). Effects of hypoxic interval training on cycling performance. Medicine and Science in Sports and Exercise, 37(1), 138–146. 15632680 10.1249/01.mss.0000150077.30672.88

[phy270349-bib-0045] Sá Filho, A. S. , Bittar, R. D. , Inacio, P. A. , Sales, M. M. , Mello, J. B. , Oliveira‐Silva, I. , Leonardo, P. S. , Chiappa, G. R. , Lopes‐Martins, R. B. , & Santos, T. M. (2024). High‐Intensity Interval Training in Different Slopes on Aerobic Performance: A Randomized Controlled Trial.

[phy270349-bib-0046] Schabort, E. J. , Killian, S. C. , St Clair Gibson, A. , Hawley, J. A. , & Noakes, T. D. (2000). Prediction of triathlon race time from laboratory testing in national triathletes. Medicine & Science in Sports & Exercise, 32(4), 844–849. 10.1097/00005768-200004000-00018 10776905

[phy270349-bib-0047] Seiler, K. S. , & Kjerland, G. O. (2006). Quantifying training intensity distribution in elite endurance athletes: Is there evidence for an “optimal” distribution? Scandinavian Journal of Medicine and Science in Sports, 16(1), 49–56. 10.1111/j.1600-0838.2004.00418.x 16430681

[phy270349-bib-0048] Sherrington, C. , Herbert, R. D. , Maher, C. G. , & Moseley, A. M. (2000). PEDro. A database of randomized trials and systematic reviews in physiotherapy. Manual Therapy, 5(4), 223–226. 10.1054/math.2000.0372 11052901

[phy270349-bib-0049] Sinex, J. A. , & Chapman, R. F. (2015). Hypoxic training methods for improving endurance exercise performance. Journal of Sport and Health Science, 4(4), 325–332. 10.1016/j.jshs.2015.07.005

[phy270349-bib-0050] Snell, P. G. , & Mitchell, J. H. (1984). The role of maximal oxygen uptake in exercise performance. Clinics in Chest Medicine, 5(1), 51–62. 6723243

[phy270349-bib-0051] Spencer, M. R. , & Gastin, P. B. (2001). Energy system contribution during 200‐ to 1500‐m running in highly trained athletes. Medicine and Science in Sports and Exercise, 33(1), 157–162. 10.1097/00005768-200101000-00024 11194103

[phy270349-bib-0052] Tatte, S. , Sharma, A. , Adkitte, R. , & Khan, Y. (2022). Eicacy of high‐altitude training on running performance in elite Indian long‐distance runners. Saudi Journal of Sports Medicine, 22(3), 107–112.

[phy270349-bib-0053] van der Zwaard, S. , Brocherie, F. , & Jaspers, R. T. (2021). Under the Hood: Skeletal muscle determinants of endurance performance. Frontiers in Sports and Active Living, 3, 719434. 10.3389/fspor.2021.719434 34423293 PMC8371266

[phy270349-bib-0054] Wahl, P. , Hägele, M. , Zinner, C. , Bloch, W. , & Mester, J. (2010). High intensity training (HIT) for the improvement of endurance capacity of recreationally active people and in prevention & rehabilitation (High Intensity Training (HIT) für die Verbesserung der Ausdauerleistungsfähigkeit von Normalpersonen und im Präventions‐ & Rehabilitationsbereich). Wiener Medizinische Wochenschrift, 160(23–24), 627–636. 10.1007/s10354-010-0857-3 21221914

[phy270349-bib-0055] Wen, D. , Utesch, T. , Wu, J. , Robertson, S. , Liu, J. , Hu, G. , & Chen, H. (2019). Effects of different protocols of high intensity interval training for VO2max improvements in adults: A meta‐analysis of randomised controlled trials. Journal of Science and Medicine in Sport, 22(8), 941–947. 10.1016/j.jsams.2019.01.013 30733142

[phy270349-bib-0056] Westmacott, A. , Sanal‐Hayes, N. E. M. , McLaughlin, M. , Mair, J. L. , & Hayes, L. D. (2022). High‐intensity interval training (HIIT) in hypoxia improves maximal aerobic capacity more than HIIT in Normoxia: A systematic review, meta‐analysis, and meta‐regression. International Journal of Environmental Research and Public Health, 19(21), 14261. 10.3390/ijerph192114261 36361141 PMC9658399

[phy270349-bib-0057] Yu, Q. , Kong, Z. , Zou, L. , Chapman, R. , Shi, Q. , & Nie, J. (2023). Comparative efficacy of various hypoxic training paradigms on maximal oxygen consumption: A systematic review and network meta‐analysis. Journal of Exercise Science and Fitness, 21(4), 366–375. 10.1016/j.jesf.2023.09.001 37854170 PMC10580050

[phy270349-bib-0058] Żebrowska, A. , Jastrzębski, D. , Sadowska‐Krępa, E. , Sikora, M. , & Di Giulio, C. (2019). Comparison of the effectiveness of high‐intensity interval training in hypoxia and Normoxia in healthy male volunteers: A pilot study. BioMed Research International, 2019, 7315714. 10.1155/2019/7315714 31662994 PMC6778879

