# Supplementary material for: Methodological and aerobic capacity adaptations of high‐intensity interval training at different altitudes in distance runners: A comprehensive meta‐analysis
Source: Physiol Rep. 2025 May 1;13(9):e70349. doi: 10.14814/phy2.70349 (PMC12045700; doi:10.14814/phy2.70349)
Supplement: Supplementary file 1 — Appendix S1. [file PHY2-13-e70349-s001.zip › PHYSREP-2024-12-842-file002.docx]

**Supplementary (S) Tables**

**Table S1** Summary of searched databases and keywords used in the review.

| **Heat** | **Search term** | **PubMed** | **Cochrane** | **ScienceDirect** | **Europe PMC** | **ProQuest** | **Total** |
| --- | --- | --- | --- | --- | --- | --- | --- |
| 1 | [High-intensity interval training] and [altitude or hypoxic training] | 64 | 4 | 172 | 290 | 80 | 610 |
| 2 | [sprint interval training] and [altitude or hypoxic training] | 67 | 0 | 75 | 65 | 61 | 268 |
| 3 | [Repeated sprint training] and [altitude or hypoxic training] | 67 | 0 | 81 | 88 | 69 | 305 |
| Total | | 198 | 4 | 328 | 443 | 210 | 1,183 |

**Table S2** Studies excluded from the review due to reasons.

| **No** | **Study** | **Exclusion reason** |
| --- | --- | --- |
| 1 | Abouzeid et al., 2023 | A mix of participants from cycling, swimming and running |
| 2 | Adams et al., 1975 | Below the required intensity training |
| 3 | Bailey et al., 1998 | No primary measurement |
| 4 | Burtscher et al., 1996 | Volunteer runners and mix of base, interval (speed & hill) training |
| 5 | Chapman et al., 1998 | Repeated study by Levine & Stray-Gundersen, 1997 |
| 6 | De Smet et al., 2017 | Active Participants are not specified |
| 7 | Holliss et al., 2014 | No primary measurement and Training intensity is below the required |
| 8 | Katayama et al., 2003 | Hypoxic exposure at rest |
| 9 | Park et al., 2019 | Continuous and HIIT were given for both groups at normoxia |
| 10 | Ponsot et al., 2006 | Repeated study by Dufour et al., 2006 |
| 11 | Porcari et al., 2016 | Volunteers and sport events not identified clearly |
| 12 | Robach et al., 2014 | No primary measurement and Training intensity is below the required |
| 13 | Stray-Gundersen et al., 2001 | No comparable group |
| 14 | Yi et al., 2024 | No primary measurement |
| 15 | Zoll et al., 2006 | repeated study by Dufour et al., 2006 |

**References**

Abouzeid, N., et al., *Eight Weeks of High-Intensity Interval Training Using Elevation Mask May Improve Cardiorespiratory Fitness, Pulmonary Functions, and Hematological Variables in University Athletes.* International Journal of Environmental Research and Public Health, 2023. 20(4): p. 3533.

Adams, W.C., et al., *Effects of equivalent sea-level and altitude training on VO2max and running performance.* J Appl Physiol, 1975. 39(2): p. 262-6.

Bailey, D.M., et al., *Implications of moderate altitude training for sea-level endurance in elite distance runners.* Eur J Appl Physiol Occup Physiol, 1998. 78(4): p. 360-8.

Burtscher, M., et al., *Benefits of training at moderate altitude versus sea level training in amateur runners.* Eur J Appl Physiol Occup Physiol, 1996. 74(6): p. 558-63.

De Smet, S., et al., *Physiological Adaptations to Hypoxic vs. Normoxic Training during Intermittent Living High.* Front Physiol, 2017. 8: p. 347.

Holliss, B.A., et al., *Eight weeks of intermittent hypoxic training improves submaximal physiological variables in highly trained runners.* J Strength Cond Res, 2014. 28(8): p. 2195-203.

Katayama, K., et al., *Intermittent hypoxia improves endurance performance and submaximal exercise efficiency.* High Alt Med Biol, 2003. 4(3): p. 291-304.

Levine, B.D. and J. Stray-Gundersen, *"Living high-training low": effect of moderate-altitude acclimatization with low-altitude training on performance.* J Appl Physiol (1985), 1997. 83(1): p. 102-12.

Park, H.Y., W. Park, and K. Lim, *Living High-Training Low for 21 Days Enhances Exercise Economy, Hemodynamic Function, and Exercise Performance of Competitive Runners.* J Sports Sci Med, 2019. 18(3): p. 427-437.

Ponsot, E., et al., *Exercise training in normobaric hypoxia in endurance runners. II. Improvement of mitochondrial properties in skeletal muscle.* J Appl Physiol (1985), 2006. 100(4): p. 1249-57.

Porcari, J.P., et al., *Effect of Wearing the Elevation Training Mask on Aerobic Capacity, Lung Function, and Hematological Variables.* J Sports Sci Med, 2016. 15(2): p. 379-86.

Robach, P., et al., *Hypoxic training: effect on mitochondrial function and aerobic performance in hypoxia.* Med Sci Sports Exerc, 2014. 46(10): p. 1936-45.

Stray-Gundersen, J., R.F. Chapman, and B.D. Levine, *"Living high-training low" altitude training improves sea level performance in male and female elite runners.* J Appl Physiol (1985), 2001. 91(3): p. 1113-20.

Yi, L., et al., *Effects of three weeks base training at moderate simulated altitude with or without hypoxic residence on exercise capacity and physiological adaptations in well-trained male runners.* PeerJ, 2024. 12: p. e17166.

Zoll, J., et al., *Exercise training in normobaric hypoxia in endurance runners. III. Muscular adjustments of selected gene transcripts.* J Appl Physiol (1985), 2006. 100(4): p. 1258-66.
