# Supplementary material for: Methodological and aerobic capacity adaptations of high‐intensity interval training at different altitudes in distance runners: A comprehensive meta‐analysis
Source: Physiol Rep. 2025 May 1;13(9):e70349. doi: 10.14814/phy2.70349 (PMC12045700; doi:10.14814/phy2.70349)
Supplement: Supplementary file 1 — Appendix S1. [file PHY2-13-e70349-s001.zip › PHYSREP-2024-12-842-file003.docx]

Table S3 PRISMA checklist for the Methodological and Aerobic Capacity Adaptations of High-Intensity Interval Training at Different Altitudes in Distance Runners: A Comprehensive Meta-Analysis

| Section/Topic | |  | Checklist item | Reported on page |
| --- | --- | --- | --- | --- |
| **TITLE** | | | |  |
| Title | 1 | | Identify the report as a systematic review and meta-analysis. | 1 |
| **ABSTRACT** | | | |  |
| Structured summary | 2 | | Structured summary including, as applicable:  **Background:** main objectives  **Methods:** data sources; study eligibility criteria, participants, and interventions; study appraisal; and synthesis methods, such as network meta-analysis.  **Results:** number of studies and participants identified; summary estimates with corresponding confidence intervals; treatment rankings may also be discussed. Authors may choose to summarize pairwise comparisons against a chosen treatment included in their analyses for brevity.  **Conclusions:** limitations; conclusions and implications of findings.  **Other:** systematic review registration number with registry name. | 1 |
| **INTRODUCTION** | | | |  |
| Rationale | 3 | | Describe the rationale for the review in the context of already known and why the meta-analysis has been conducted.. | 1-3 |
| Objectives | 4 | | Provide an explicit statement of objectives and questions being addressed, using PICOS (participants, interventions, comparisons, outcomes, and study design). | 3 |
| **METHODS** |  | |  |  |
| Registration and protocol | 5 | | Provide registration information for the review, including register name and registration number. | 3 |
|  |  | | Indicate where the review protocol can be accessed, or state that a protocol was not prepared. |  |
|  |  | | Indicate where the review protocol can be accessed, or state that a protocol was not prepared. |  |
| Eligibility criteria | 6 | | Specify study characteristics (e.g., PICOS, length of follow-up) and report characteristics (e.g., years considered, language, publication status) used as criteria for eligibility, giving rationale. Clearly describe eligible treatments included in the treatment network, and note whether any have been clustered or merged into the same node (with justification). | 3-4 |
| Information sources | 7 | | Specify all databases, registers, websites, organisations, reference lists and other sources searched or consulted to identify studies. Specify the date when each source was last searched or consulted. | 4 |
| Search strategy | 8 | | Present the full search strategies for all databases, registers and websites, including any filters and limits used. | 4 |
| Study selection process | 8 | | Specify the methods used to decide whether a study met the inclusion criteria of the review, including how many reviewers screened each record and each report retrieved, whether they worked independently, and if applicable, details of automation tools used in the process. | 4 |
| Data collection process | 9 | | Specify the methods used to collect data from reports, including how many reviewers collected data from each report, whether they worked independently, any processes for obtaining or confirming data from study investigators, and if applicable, details of automation tools used in the process. | 4 |
| Data items | 10 | | Describe any assumptions made about any missing or unclear information. | 4 |
| Study risk of bias within study | 11 | | Specify the methods used to assess risk of bias in the included studies, including details of the tool(s) used, how many reviewers assessed each study and whether they worked independently, and if applicable, details of automation tools used in the process. | 4 |
| Effect measures | 12 | | Specify for each outcome the effect measure(s) (e.g. risk ratio, mean difference) used in the synthesis or presentation of results. | 5 |
| Synthesis methods | 13 | | Describe the processes used to decide which studies were eligible for each synthesis (e.g. tabulating the study intervention characteristics and comparing against the planned groups for each synthesis (item #5)). | 5 |
|  |  | | Describe any methods required to prepare the data for presentation or synthesis, such as handling of missing summary statistics, or data conversions. | 5 |
|  |  | | Describe any methods used to tabulate or visually display results of individual studies and syntheses. | NA |
|  |  | | Describe any methods used to synthesize results and provide a rationale for the choice(s). If meta-analysis was performed, describe the model(s), method(s) to identify the presence and extent of statistical heterogeneity, and software package(s) used. | 5 |
|  |  | | Describe any methods used to explore possible causes of heterogeneity among study results (e.g. subgroup analysis, meta-regression). | 5 |
|  |  | | Describe any sensitivity analyses conducted to assess robustness of the synthesized results. | 5 |
| Reporting bias assessment | 14 | | Describe any methods used to assess risk of bias due to missing results in a synthesis (arising from reporting biases). | 6 |
| Certainty assessment | 15 | | Describe any methods used to assess certainty (or confidence) in the body of evidence for an outcome. | 6 |
| **RESULTS** | | | |  |
| Study selection | 16 | | Describe the results of the search and selection process, from the number of records identified in the search to the number of studies included in the review, ideally using a flow diagram. | 6 |
|  |  | | Cite studies that might appear to meet the inclusion criteria, but which were excluded, and explain why they were excluded. | 5 |
| Study characteristics | 17 | | Cite each included study and present its characteristics. | 7 |
| Risk of bias within studies | 18 | | Present assessments of risk of bias for each included study. | 8 |
| Results of individual studies | 19 | | For all outcomes, present, for each study: (a) summary statistics for each group (where appropriate) and (b) an effect estimate and its precision (e.g. confidence/credible interval), ideally using structured tables or plots. | 8 |
| Syntheses of results | 20 | | For each synthesis, briefly summarise the characteristics and risk of bias among contributing studies. | - |
|  |  | | Present results of all statistical syntheses conducted. If meta-analysis was done, present for each the summary estimate and its precision (e.g. confidence/credible interval) and measures of statistical heterogeneity. If comparing groups, describe the direction of the effect. | 8 |
|  |  | | Present results of all investigations of possible causes of heterogeneity among study results. | 12-14 |
|  |  | | Present results of all sensitivity analyses conducted to assess the robustness of the synthesized results. | 13 |
| Reporting biases | 21 | | Present assessments of risk of bias due to missing results (arising from reporting biases) for each synthesis assessed. | 14 |
| Certainty of evidence | 22 | | Present assessments of certainty (or confidence) in the body of evidence for each outcome assessed. | - |
| **DISCUSSION** | | | |  |
| Evidence summary | 23 | | Provide a general interpretation of the results in the context of other evidence. | 14-15 |
| limitations |  | | Discuss any limitations of the evidence included in the review. | 15 |
|  |  | | Discuss any limitations of the review processes used. | 15 |
|  |  | | Discuss implications of the results for practice, policy, and future research. | 15-16 |
| Conclusions | 24 | | Provide a general interpretation of the results in the context of the evidence, and implications for future research. | 15 |
| Support | 25 | | Describe sources of financial or non-financial support for the review, and the role of the funders or sponsors in the review. | None |
| Competing interests | 26 | | Declare any competing interests of review authors. | 16 |
| Availability of data, code and other materials | 27 | | Report which of the following are publicly available and where they can be found: template data collection forms; data extracted from included studies; data used for all analyses; analytic code; any other materials used in the review. | 16 |
